# Substituted aminopyrimidine protein kinase B (PknB) inhibitors show activity against *Mycobacterium tuberculosis*

**DOI:** 10.1016/j.bmcl.2012.02.107

**Published:** 2012-05-01

**Authors:** Timothy M. Chapman, Nathalie Bouloc, Roger S. Buxton, Jasveen Chugh, Kathryn E.A. Lougheed, Simon A. Osborne, Barbara Saxty, Stephen J. Smerdon, Debra L. Taylor, David Whalley

**Affiliations:** a Centre for Therapeutics Discovery, MRC Technology, 1-3 Burtonhole Lane, Mill Hill, London NW7 1AD, UK; b Division of Mycobacterial Research, MRC National Institute for Medical Research, The Ridgeway, Mill Hill, London NW7 1AA, UK; c Division of Molecular Structure, MRC National Institute for Medical Research, The Ridgeway, Mill Hill, London NW7 1AA, UK

**Keywords:** Protein kinase B, *Mycobacterium tuberculosis*, Aminopyrimidine, Structure–activity relationship

## Abstract

A high-throughput screen against PknB, an essential serine–threonine protein kinase present in *Mycobacterium tuberculosis* (*M. tuberculosis*), allowed the identification of an aminoquinazoline inhibitor which was used as a starting point for SAR investigations. Although a significant improvement in enzyme affinity was achieved, the aminoquinazolines showed little or no cellular activity against *M. tuberculosis*. However, switching to an aminopyrimidine core scaffold and the introduction of a basic amine side chain afforded compounds with nanomolar enzyme binding affinity and micromolar minimum inhibitory concentrations against *M. tuberculosis*. Replacement of the pyrazole head group with pyridine then allowed equipotent compounds with improved selectivity against a human kinase panel to be obtained.

## Graphical abstract

**Figure:**
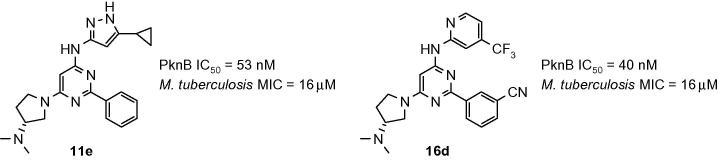

More than 14 million people worldwide suffer from tuberculosis (TB) which in 2009 resulted in an estimated 1.7 million deaths.^1^ In particular, the increasing prevalence of multi-drug resistant strains means that novel therapeutic approaches are urgently needed. PknB is one of 11 serine–threonine protein kinases (STPKs) found in *Mycobacterium tuberculosis* (*M. tuberculosis*) and has been shown to be essential for bacterial growth,^2^ and therefore inhibition of PknB represents a potential therapeutic approach for the treatment of TB. PknB is an attractive target as the kinase domain contains less than 30% similarity with eukaryotic kinases, which offers promise for achieving selectivity for the mycobacterial kinase over those of the human host. *M. tuberculosis* is unusual amongst bacteria in having a higher number of STPKs in comparison to the more common two-component signalling systems,^3^ and of the other STPKs present, PknA has also been found to be essential while PknG has been reported to play a crucial role in the survival of mycobacteria within macrophages.^4^ Previous studies have focused on the potential of PknB and PknG as drug targets in *M. tuberculosis*, although the majority have reported significantly lower activity against whole cells than against the purified enzymes.^2,4,5^ This difference may be due in part to the nature of the TB cell wall, which is recognised as difficult to permeate, particularly due to its unique mycolic acid layer.^6^

A high throughput screen of our compound collection against the isolated PknB enzyme was performed through measuring the effect on the in vitro phosphorylation of the substrate GarA,^7^ and this identified compound **1** (Fig. 1) as a 1.35 μM inhibitor of PknB, which formed the starting point for our chemistry programme.

Initial SAR exploration around **1** was performed via synthetic access from 2,4-dichloroquinazoline **3** (Scheme 1). Introduction of the aminopyrazole was performed by nucleophilic substitution at the 4-position, followed by either a Suzuki coupling to introduce an aryl group or a second nucleophilic substitution to introduce a second amino substituent at the 2-position.

Replacement of the pyrazole methyl substituent with cyclopropyl gave a fourfold improvement in binding affinity (Table 1, **5a**). The 4-pyridyl group could also be substituted by other aryl groups such as 4-fluorophenyl or phenyl while maintaining comparable potency (**5b**–**5e**). Alkyl amines were also tolerated at this position, with cycloalkyl groups such as the cyclohexyl derivative **5f** giving the best IC_50_. However, the introduction of highly polar or charged groups was not well tolerated at this position and led to a significant loss in potency. Screening of these compounds against *M. tuberculosis* in an Alamar blue cellular assay^8^ revealed that they possessed weak or no cellular efficacy. Measured log *D* values for these compounds were typically in excess of 4, and measurement of their plasma–protein binding (ppb) revealed that they were highly bound (>99%), leaving only a small free fraction available to bind to the enzyme. Furthermore, the physical properties of these compounds were not considered to be well disposed for crossing the TB cell wall, as its mycolic acid layer contains barriers to the passage of hydrophobic as well as hydrophilic molecules.^6^

In order to improve the physical properties of the compounds with the aim of improving their cellular activity, it was decided to reduce the log *D* of the compounds and explore the effect of this in advancing the SAR. The quinazoline core was switched to a pyrimidine, and while retaining the cyclopropylaminopyrazole at the 4-position, a range of substituents including aryl and amino groups were introduced at the 2-position to allow exploration of the SAR (Scheme 2). This afforded compounds with improved potency against the enzyme (Table 2), and amongst the aryl variants, the 3-sulfonylphenyl compound **8d** and 3-cyanophenyl derivative **8f** showed the highest affinities at 86 and 87 nM, respectively. For the amino compounds, in addition to the cyclohexyl and cyclopentyl examples **8h** and **8n** at 84 and 115 nM, the substituted phenylethylamines **8k** and **8l** showed promising enzyme affinities of 74 and 64 nM, respectively. These compounds also demonstrated improved efficacy against *M. tuberculosis* although this was still relatively weak, with the majority of minimum inhibitory concentrations (MICs) falling in the 63–250 μM range. The most active example was the 3-sulfonamidophenyl variant **8c** at 31 μM.

Further improvements to the physical properties of the compounds were sought, and a basic amine side chain was introduced to give compounds with a lower log *D*. The 6-position was chosen as a suitable point for appending this side-chain as modelling studies suggested that this region of the molecule was solvent exposed, so would be amenable to the introduction of this group. A range of basic side chains were introduced into this position, initially with a phenyl group fixed at the 2-position (Scheme 3). The products, shown in Table 3 demonstrated sub-100 nM enzyme potency and generally improved MIC values, and compound **11e** containing the (*R*)-3-dimethylaminopyrrolidine basic side-chain displayed an IC_50_ of 53 nM against the enzyme and a 16 μM MIC against *M. tuberculosis*. Oxygen-linked basic side chains were also well tolerated, for example compound **11f**, which showed an IC_50_ of 56 nM against the enzyme and 31 μM MIC against the *Mycobacterium*.

Considering the enhanced enzyme binding affinity that had resulted from the presence of a 3-cyano or 3-sulfonyl group on the phenyl ring in the disubstituted variants (Table 2), these substituents were introduced into the trisubstituted pyrimidines aiming for a corresponding increase in enzyme potency and activity against *M. tuberculosis*. Starting with 4,6-dichloro-2-(methylthio)pyrimidine **12**, the cyclopropylaminopyrazole group and the basic amine side-chain were sequentially introduced under nucleophilic displacement conditions. This was followed by a Liebeskind–Srogl coupling^9^ under microwave heating on the *S*-methyl pyrimidine **14** to install the desired aryl group (Scheme 4). However, the resultant compounds displayed modest gains in enzyme affinity (approximately twofold for **15b** and **15c**) and no significant improvement in activity against *M. tuberculosis* (Table 4), which remained between 31 and 63 μM.

Despite the general improvement in MIC values obtained through introduction of the basic side chain to the pyrimidine core, efficacy against *M. tuberculosis* remained weaker than was desired. In an effort to improve the cellular activity, it was decided to replace the aminopyrazole head group with an aminopyridine group. This allowed the hydrogen bond donor count to be reduced, which was considered desirable for improving permeability through the *M. tuberculosis* cell wall. It also offered the potential for improved kinase selectivity by reducing the reliance on the binding energy gained at the kinase hinge region. These compounds were prepared following a similar sequence to that described in Scheme 4, replacing the aminopyrazole with the appropriate aminopyridine but using a palladium-catalysed amination for introduction of the aminopyridine instead of nucleophilic displacement. Initial variants showed a significant loss in binding affinity, but further exploration of the SAR revealed that pyridines carrying a substituent at the 4-position were optimal for enzyme inhibition and this allowed the IC_50_ to be restored to the double-digit nanomolar range (Table 5). However, in common with the aminopyrazoles, MIC values against *M. tuberculosis* remained in the micromolar region. Compound **16c** displayed the most promising activity with a MIC of 8 μM.

The selectivity of the inhibitors was examined against other *M. tuberculosis* kinases and this revealed that they typically showed cross-affinity with PknF, although they displayed only modest inhibition of PknG. Aminopyrimidines featuring appended pyrazole groups have been reported in the literature as inhibitors of a number of human kinases,^10^ so achieving selectivity in these compounds was therefore a key consideration and their kinase selectivity against a 76-member human kinase panel was examined. Pleasingly compound **16c**, containing the aminopyridine head group motif, showed an improvement in selectivity relative to **11e**, which contains the aminopyrazole head group (Fig. 2). This is consistent with an expected gain in selectivity that may occur through reducing the number of H-bonds that can be formed with the kinase hinge region. Docking studies were performed using Glide™ (Schrödinger Inc.) on both the aminopyrazole and aminopyridine head group variants using the PknB crystal structure (ref: 2FUM)^12^ from the PDB, and the predicted binding modes of compounds **15c** and **16d** are shown in Figure 3. It can be seen that the aminopyrazoles can form up to 3 hydrogen bonds with the hinge region of the kinase, and the appended cyclopropyl group can form a face-on lipophilic interaction with the methionine gatekeeper residue. The cyano group on the phenyl ring points in the direction of a lysine residue. By contrast, in the aminopyridine variants two hydrogen bonds with the hinge region can be formed, and the 4-substituent on the pyridine head group is orientated in the direction of the gatekeeper.

In summary, the starting point HTS hit was optimised for potency against PknB, and initial modifications showed promising gains in enzyme binding affinity but little or no cellular activity against *M. tuberculosis*. Switching from the quinazoline to a pyrimidine core and introduction of a basic side chain improved the physical properties of the molecules and resulted in improved cellular activities, in the double-digit micromolar region. While further optimisation gave improvements in enzyme binding affinity, this did not translate into improved efficacy against *M. tuberculosis*. Replacement of the aminopyrazole head group with an aminopyridine led to an initial loss in binding affinity, but this was regained following optimisation, and selectivity against a human kinase panel was improved. Although the best of these compounds still only showed whole cell MICs in the 10 μM region, this level of activity is commensurate with that reported for a number of other heterocyclic compounds which have shown activity against *M. tuberculosis*.^13^ Possible reasons for the significant difference in enzyme and whole cell activity were investigated, including poor cell wall permeability, the role of efflux pumps, protein binding in the assay media and inhibitor specificity, and this work is described in a separate paper.^8^

## Figures and Tables

**Figure 1:**
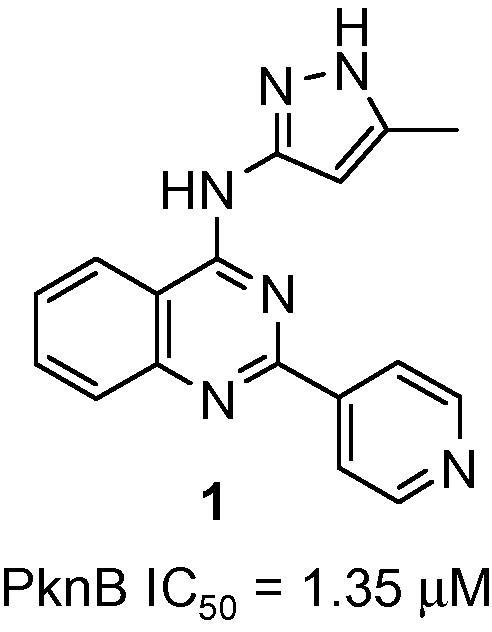
HTS hit **1**.

**Figure 2:**
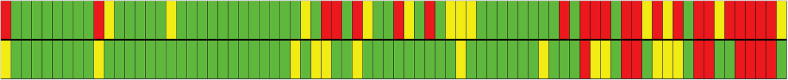
Kinase selectivity heat map comparing compounds **11e** (top) and **16c** (bottom) against a 76-member human kinase panel, screened at 10 μM inhibitor concentration. [Key: red: >90% inhibition; yellow: 70–90% inhibition; green: <70% inhibition.^11^]

**Figure 3:**
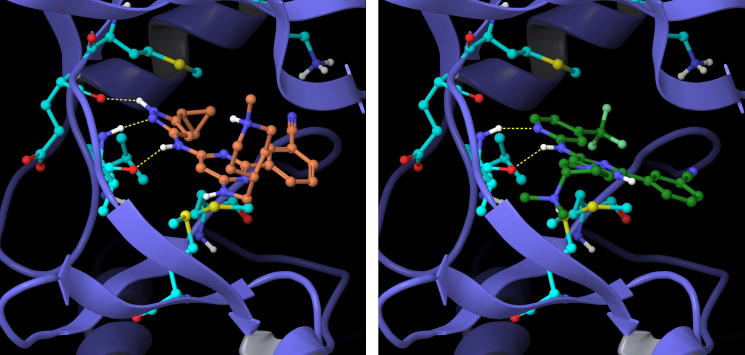
Example aminopyrazole **15c** (left) and aminopyridine **16d** (right) head group compounds docked into the PknB active site.

**Scheme 1:**
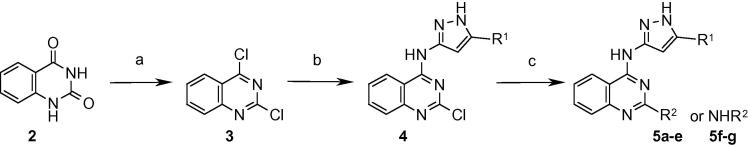
Reagents and conditions: (a) POCl_3_, *N*,*N*-dimethylaniline, reflux; (b) 3-amino-5-R^1^-pyrazole, EtOH, 20 °C; (c) R^2^B(OH)_2_, Pd(PPh_3_)_2_Cl_2_, Et_3_N, MeOH, microwave, 140 °C or R^2^NH_2_, EtOH, microwave, 150 °C.

**Scheme 2:**
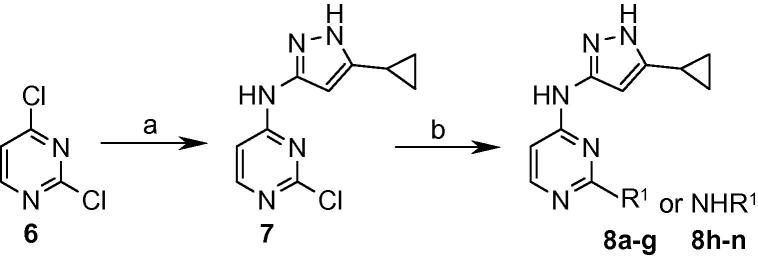
Reagents and conditions: (a) 3-Amino-5-cyclopropylpyrazole, DIPEA, *i*-PrOH, 50 °C; (b) R^1^B(OH)_2_, Pd(PPh_3_)_2_Cl_2_, Et_3_N, MeOH, microwave, 150 °C or R^1^NH_2_, DIPEA, *i*-PrOH, microwave, 170 °C.

**Scheme 3:**
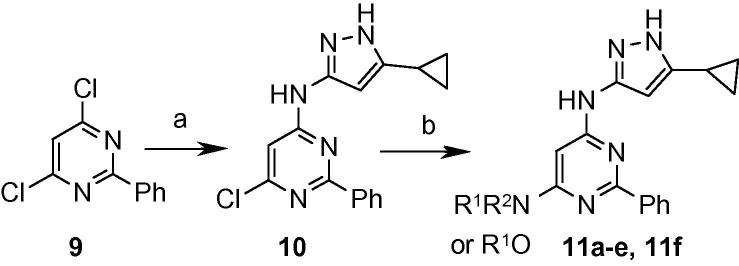
Reagents and conditions: (a) 3-Amino-5-cyclopropylpyrazole, DIPEA, NaI, DMA, 100 °C; (b) R^1^R^2^NH, *n*-BuOH, microwave, 150–170 °C or R^1^OH, *t*-BuOK, THF, microwave, 160 °C.

**Scheme 4:**
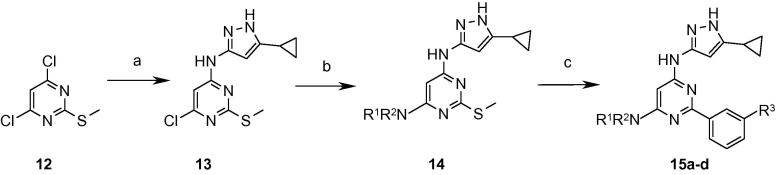
Reagents and conditions: (a) 3-Amino-5-cyclopropylpyrazole, Et_3_N, NaI, DMA, microwave, 150 °C; (b) R^1^R^2^NH, *n*-BuOH, microwave, 170 °C; (c) R^3^PhB(OH)_2_, Pd(PPh_3_)_4_, copper(I) thiophene carboxylate, dioxane, microwave, 150 °C.

**Table 1:** SAR of disubstituted quinazolines

Compound	R^1^	R^2^ or NHR^2^	PknB IC_50_/μM^a^
**5a**			0.322
**5b**			0.453
**5c**			1.162
**5d**			0.223
**5e**			0.440
**5f**			0.107
**5g**			0.428

a Assay conditions are described in Ref. 8.

**Table 2:** SAR of disubstituted pyrimidines

Compound	R^1^ or NHR^1^	PknB IC_50_/μM^a^	*M. tuberculosis* MIC/μM^a^
**8a**		0.375	>500
**8b**		0.384	63
**8c**		0.393	31
**8d**		0.086	63
**8e**		0.241	63
**8f**		0.087	125
**8g**		0.202	125
**8h**		0.084	63
**8i**		0.354	63
**8j**		0.715	125
**8k**		0.074	63
**8l**		0.064	125
**8m**		0.217	250
**8n**		0.115	63

a Assay conditions are described in Ref. 8.

**Table 3:** SAR of trisubstituted pyrimidines

Compound	NR^1^R^2^ or OR^1^	PknB IC_50_/μM^a^	*M. tuberculosis* MIC/μM^a^	*m* Log *D*^b^
**11a**		0.088	125	3.7
**11b**		0.121	63	3.6
**11c**		0.055	31	2.3
**11d**		0.073	31	2.3
**11e**		0.053	16	3.8
**11f**		0.056	31	3.4

a Assay conditions are described in Ref. 8.

b Log *D* values were measured by BioFocus.

**Table 4:** SAR of trisubstituted pyrimidines with functionalised phenyl ring portion

Compound	NR^1^R^2^	R^3^	PknB IC_50_/μM^a^	*M. tuberculosis* MIC/μM^a^
**15a**		CN	0.056	63
**15b**		CN	0.039	63
**15c**		CN	0.023	31
**15d**		SO_2_Me	0.040	63

a Assay conditions are described in Ref. 8.

**Table 5:** SAR of trisubstituted pyrimidines containing pyridine head group

**Figure:**
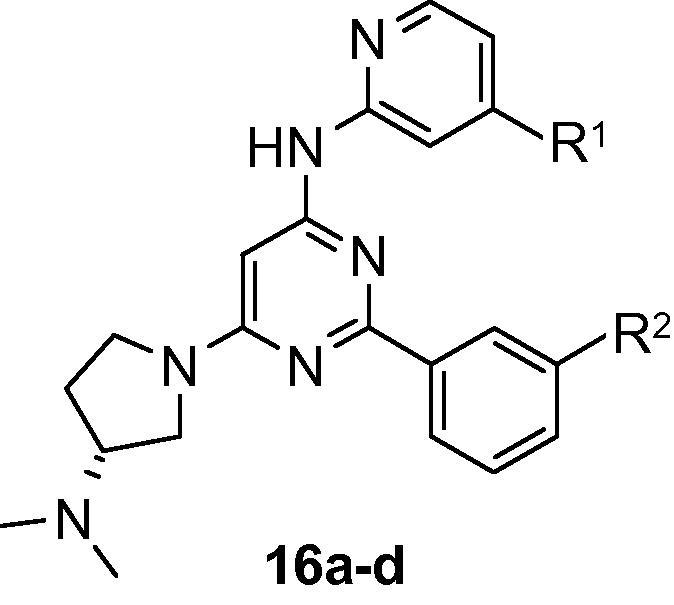

Compound	R^1^	R^2^	PknB IC_50_/μM^a^	*M. tuberculosis* MIC/μM^a^
**16a**	H	CN	1.99	31
**16b**	OMe	H	1.13	31
**16c**	Cl	H	0.176	8
**16d**	CF_3_	CN	0.040	16

a Assay conditions are described in Ref. 10.
